# TREatment of ATopic eczema (TREAT) Registry Taskforce: protocol for an international Delphi exercise to identify a core set of domains and domain items for national atopic eczema registries

**DOI:** 10.1186/s13063-016-1765-7

**Published:** 2017-02-27

**Authors:** Louise A. A. Gerbens, Aaron E. Boyce, Dmitri Wall, Sebastien Barbarot, Richard J. de Booij, Mette Deleuran, Maritza A. Middelkamp-Hup, Amanda Roberts, Christian Vestergaard, Stephan Weidinger, Christian J. Apfelbacher, Alan D. Irvine, Jochen Schmitt, Paula R. Williamson, Phyllis I. Spuls, Carsten Flohr

**Affiliations:** 1 Department of Dermatology, Academic Medical Centre, University of Amsterdam, Meibergdreef 9, 1105 AZ Amsterdam, The Netherlands; 2 Unit for Population-Based Dermatology Research, St. John’s Institute of Dermatology, Guy’s and St Thomas’ NHS Foundation Trust and King’s College London, London, UK; 3 Department of Paediatric Dermatology, Our Lady’s Children’s Hospital, Crumlin, Dublin, Ireland; 4 Irish Skin Foundation, Dublin, Ireland; 5 Department of Dermatology, Nantes University Hospital, Nantes, France; 6 Department of Dermatology and Venereology, Aarhus University Hospital, Aarhus, Denmark; 7 Nottingham Support Group for Carers of Children with Eczema, Nottingham, UK; 8 Department of Dermatology and Allergy, University Hospital Schleswig-Holstein, Campus Kiel, Kiel, Germany; 9 Medical Sociology, Institute of Epidemiology and Preventive Medicine, University of Regensburg, Regensburg, Germany; 10 Department of Clinical Medicine, Trinity College Dublin, Dublin, Ireland; 11 National Children’s Research Centre, Dublin, Ireland; 12 Center for Evidence-based Healthcare, Medizinische Fakultät Carl Gustav Carus, TU Dresden, Dresden, Germany; 13 University Allergy Center, University Hospital Carl Gustav Carus Dresden, Dresden, Germany; 14 MRC North West Hub for Trials Methodology Research, Department of Biostatistics, University of Liverpool, Liverpool, UK

**Keywords:** Atopic eczema, Atopic dermatitis, Delphi, Consensus methods, Patient registries, Disease registries, Core set, Daily practice data, Immunomodulatory therapies, Interoperability

## Abstract

**Background:**

Patients with moderate-to-severe atopic eczema (AE) often require photo- or systemic immunomodulatory therapies to induce disease remission and maintain long-term control. The current evidence to guide clinical management is small, despite the frequent and often off-label use of these treatments. Registries of patients on photo- and systemic immunomodulatory therapies could fill this gap, and the collection of a core set concerning these therapies in AE will allow direct comparisons across registries as well as data sharing and pooling.

Using an eDelphi approach, the international TREatment of ATopic eczema (TREAT) Registry Taskforce aims to seek consensus between key stakeholders internationally on a core set of domains and domain items for AE patient registries with a research focus that collect data of children and adults on photo- and systemic immunomodulatory therapies.

**Methods/design:**

Participants from six stakeholder groups will be invited: doctors, nurses, non-clinical researchers, patients, as well as industry and regulatory body representatives. The eDelphi will comprise three sequential online rounds, requesting participants to rate the importance of each proposed domain and domain items. Participants will be able to add domains and domain items to the proposed list in round 1. A final consensus meeting will be held with representatives of each stakeholder group.

**Discussion:**

Identifying a uniform core set of domains and domain items to be captured by AE patient registries will increase the utility of individual registries, and provide greater insight into the effectiveness, safety and cost-effectiveness of photo- and systemic immunomodulatory therapies to guide clinical management across dermatology centres and country borders.

**Trial registration:**

Not applicable. This eDelphi study was registered in the Core Outcome Measures for Effectiveness Trials (COMET) database.

**Electronic supplementary material:**

The online version of this article (doi:10.1186/s13063-016-1765-7) contains supplementary material, which is available to authorized users.

## Background

Atopic eczema (AE) (synonymously ‘atopic dermatitis’) is among the most common chronic inflammatory disorders, with a lifetime prevalence of 15–30% in children and 2–10% in adults [1] and is known to give a high burden of disease to patients, their families and society [2]. Whilst most patients with AE can be treated effectively with emollients and topical anti-inflammatory agents, a significant number will require photo- or systemic immunomodulatory therapies to induce disease remission and maintain long-term control [3]. Ciclosporin is currently the only systemic treatment approved by the European Medicines Agency (EMA) as a treatment for severe AE [4]. Alternative, but off-label, systemic immunomodulatory therapies include methotrexate, azathioprine, mycophenolic acid (mycophenolate mofetil or mycophenolate sodium), systemic glucocorticosteroids, and intravenous immunoglobulin. Novel agents are currently tested in clinical trials (e.g. dupilumab, lebrikizumab, apremilast, ustekinumab, and CRTH2 antagonist (chemoattractant receptor-homologous molecule expressed on Th2 cells)).

The current evidence to guide clinical management for moderate-to-severe AE originates from a small body of randomised controlled trials (RCTs) [5] and observational studies [6–8]. There are no long-term, comparative and real-life data on the effectiveness and safety of these treatments in children or adults from large-scale, multicentre cohort studies. Several scientific guidelines and a systematic review highlight these gaps [5, 9, 10] and lament the resulting lack of clear management guidance to inform clinical practice.

Nevertheless, as shown in a recent survey among over 700 dermatologists and paediatricians from eight European countries, these immunomodulatory treatments are frequently prescribed as off-label medicines in children [11]. It is likely that these treatments are used more frequently in adults, but no research has yet been performed.

The establishment of national AE patient registries with a research focus that use the same methodology would not only partially fill the gaps in the current evidence, but could also operate as a research platform to inform the design of future RCTs and basic research and serve as an example for other initiatives. Such a platform will allow the collection of prospective daily practice data on effectiveness, safety, cost-effectiveness, subgroup characteristics, quality of care and personalised medicine. Our research group, the TREatment of ATopic eczema (TREAT) Registry Taskforce, is currently developing an interoperable patient registry platform based on best-practice guidelines for this purpose [12].

Experience has shown that disease registries that were set up without an *a priori* agreed core set are likely to yield disparate datasets, hampering direct comparisons of results and data pooling [13]. To overcome this problem, an internationally agreed core set for AE patient registries is required. The European Commission funded PAtient REgistries iNiTiative Joint Action (PARENT JA) has developed methodological guidelines for the development of patient registries in cross-border settings [12] to reduce heterogeneity, enhance direct comparability of individual country data and improve data pooling between countries.

In alignment with the PARENT guidance, the objective of this eDelphi study is to reach international consensus between different stakeholders on a core set of domains and domain items (what to measure), for existing and future atopic eczema patient registries with a research focus, that collect data of children and adults on photo- and systemic immunomodulatory therapies. This protocol outlines the methodology.

## Methods/design

This study will be conducted following the recommended checklist proposed by Sinha et al. [14], of items that should be reported in studies using the Delphi technique [15]. Details of our study have been included in the Core Outcome Measures for Effectiveness Trials (COMET) database and are available at www.comet-initiative.org/studies/details/825?result=true. The Standard Protocol Items: Recommendations for Interventional Trials (SPIRIT) checklist can be found in Additional File 1.

### Study design

**Fig. 1:**
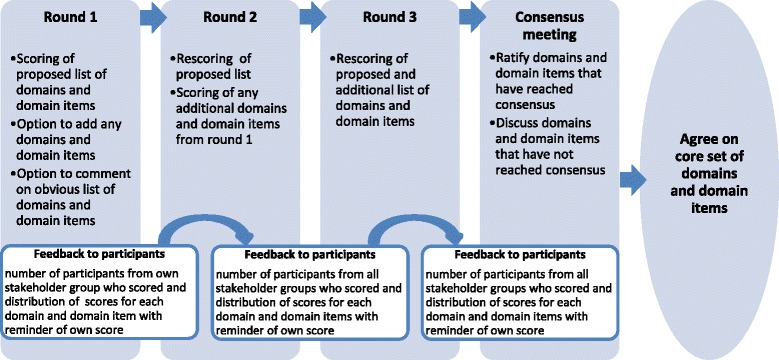
The TREatment of ATopic eczema (TREAT) Delphi exercise

To investigate domains and domain items of importance, an online Delphi exercise (further referred to as ‘eDelphi’) in combination with a consensus meeting will be conducted (Fig. 1).

The eDelphi approach comprises sequential questionnaires answered anonymously by key stakeholder groups. The answers are anonymised to avoid participants being influenced by the opinions of other group members, and thus minimise bias. After each questionnaire round, a summary of the responses is fed back to the group. Individual participants may then decide to keep their original answers or to change their opinion in the next round. Gradually, a consensus evolves as in general the range of answers decreases and the group converges towards a consensus opinion over the course of several rounds [14].

After the eDelphi exercise, a consensus meeting will be organised with representatives from each stakeholder group to resolve potentially remaining disagreements and to agree on a core set of domains and domain items.

### Participants

Representatives from six key stakeholder groups [16] will be identified and invited to participate in the consensus study:

1. Health care professionals – doctors who care for patients with moderate-to-severe AE, including doctors who are involved in AE clinical research
2. Health care professionals – nurses who care for patients with moderate-to-severe AE
3. Non-clinical researchers with a specialist and active research interest in AE, e.g. methodologists, epidemiologists, health economists
4. Patients (adult patients and carers of children or adults with AE)
5. Industry representatives from pharmaceutical companies involved in the development of systemic immunomodulatory drugs for AE
6. Regulatory body representatives from the EMA, US Food and Drug Administration (FDA) and national regulatory bodies such as the UK National Institute for Health and Care Excellence (NICE)

### Panel size and recruitment

Currently, there are no official recommendations for the number of participants to include in an eDelphi study [17]. Because of the six stakeholder groups and their possible diversity of opinions, we will recruit as many representatives for each stakeholder group as possible and from as many counties as possible.

To identify health care professionals and non-clinical researchers to participate in the eDelphi, we will approach relevant international societies registered with the International League of Dermatological Societies (ILDS) and other relevant special interest groups (e.g. the International Eczema Council) (Table 1). Societies will be asked to send out an email with the link to our eDelphi, and their members can register if they want to participate. Members who want to participate will also be asked to cascade the link to other relevant experts in the field. Patient representatives will be recruited from national eczema support groups (Table 2), again by email invitation. By sending the survey invitation to societies from different parts of the world, we aim to include patients of different skin types and cultures. Industry representatives will be invited from pharmaceutical companies that have developed systemic drugs for AE or are known to have such medications in development. Finally, representatives from regulatory bodies (i.e. EMA, FDA, national regulatory bodies) will be identified through personal contacts from Europe and the United States. The invitation letter containing an embedded link to the eDelphi questionnaire can be found in Additional file 2.

**Table 1:** List of (inter)national societies to be invited

Category	Name of society
International dermatology societies	African Association for Dermatology
	African Dermatovenereology Association
	Asian Academy of Dermatology and Venereology
	Asian Dermatological Association
	Association of French Speaking Dermatologists
	Caribbean Dermatology Association
	European Academy of Dermatology and Venereology (EADV)
	European Dermatology Forum (EDF)
	European Society for Dermatological Research (ESDR)
	European Society for Photodermatology (ESPD)
	Gulf Cooperation Council League of Dermatologists
	Ibero Latin American College of Dermatology (CILAD)
	International Forum for the Study of Itch
	International Society of Dermatology
	Pacific Dermatologic Association
	Palestinian Society of Dermatology and Venereology (PSDV)
	Pan Arab League of Dermatology
	South Asian Regional Association of Dermatologists, Venereologists and Leprologists (SARAD)
	Women’s Dermatologic Society
International dermatology special interest societies	European Dermato-Epidemiology Network (EDEN)
	European Taskforce on Atopic Dermatitis (ETFAD)
	Harmonising Outcome Measures for Eczema (HOME)
	International Eczema Council (IEC)
	International Society of Atopic Dermatitis (ISAD)
	European Registry of Psoriasis (PSONET)
Dermatology nursing societies	Australian Dermatology Nurses’ Association (ADNA)
	British Dermatological Nursing Group (BDNG)
	Dermatology Nurses Association (DNA)
	International Skin Care Nursing Group (ISNG)
Paediatric dermatology societies	British Society of Paediatric Dermatology (BSPD)
	Deutsche Gesellschaft für Kinder- und Jugendmedizin e.V. (DGKJ)
	European Society of Paediatric Dermatology (ESPD)
	French Society for Pediatric Dermatology
	International Society of Paediatric Dermatology (ISPD)
	Japanese Society of Pediatric Dermatology
	Latin American Pediatric Dermatology Society
	Nederlands-Belgische vereniging voor Kinderdermatologie (Kinderhuid)
	Society for Pediatric Dermatology (SPD)
National dermatology societies	Algerian Society of Dermatology
	American Academy of Dermatology (AAD)
	American Dermatological Association (ADA)
	Argentine Society of Dermatology (SAD)
	Asociacion Colombiana de Dermatoloiga y Cirugia Dermatologica (AsoColDerma)
	Asociacion Ecuatoriana de Dermatologia y Ciencias Afines (AEDCA)
	Asociacion Guatemalteca De Dermatologia
	Association of Dermato-Venerologists of Latvia (BADV)
	Association of Italian Clinical Dermatologists (AIDA)
	Association of the Italian Women Dermatologists (DDI)
	Association of Professors of Dermatology USA (APD)
	Australasian College of Dermatologists (ACD)
	Austrian Society for Dermatology and Venereology (OEGDV)
	Belarusian Society of Dermatovenereologists and Cosmetologists
	Belgian Society of Dermatology and Venereology
	Brazilian Society of Dermatology (SBD)
	British Association of Dermatologists (BAD)
	Bulgarian Dermatological Society
	Canadian Dermatology Association (CDA)
	Chilean Society of Dermatology and Venereology
	Chinese Society of Dermatology (CSD)
	Croatian Dermatovenerological Society of the Croatian
	Cyprus Society of Dermatology and Venereology
	Czech Academy of Dermatovenereology (CADV)
	Czech Dermatovenereology Society (CDS)
	Dansk Dermatologisk Selskab (DDS)
	Dermatologic Society of Iceland
	Dermatological Society of Malaysia (PDM)
	Dermatological Society of Mauritius
	Dermato-venereology Senegalese Society
	Dermatological Society of Singapore (DSS)
	Dermatological Society of South Africa (DSSA)
	Dermatological Society of Thailand (DST)
	Dermatovenereology Association of Turkey
	Dutch Society of Dermatology and Venereology (NVDV)
	Eczema Clinical Network New Zealand
	Egyptian Society of Aesthetic Dermatology
	Finnish Society of Dermatology (Suomen Ihotautilääkäriyhdistys) (SILY)
	French Society of Dermatology (SFD)
	Georgian Association of Dermatology and Venereology (GADV)
	German Dermatological Society (DDG)
	Hellenic Society of Dermatology and Venereology (EDAE)
	Honduran Society of Dermatology and Dermatologic Surgery
	Hong Kong College of Dermatologists (HKCD)
	Hong Kong Society of Dermatology and Venereology (HKSDV)
	Hungarian Dermatological Society (MDT)
	Indian Association of Dermatologists, Venereologists and Leprologists (IADVL)
	Indonesian Society of Dermatology and Venereology (PERDOSKI)
	Iranian Society of Dermatology
	Irish Association of Dermatologist
	Israel Society of Dermatology and Venereology (ISDV)
	Italian Association of Hospital Dermatologists (ADOI)
	Italian Society of Allergological, Occupational and Environmental Dermatology (SIDAPA)
	Italian Society of Dermatology (SIDeMaST)
	Ivoirienne Society of Dermatology and Venereology (SIDV)
	Japanese Dermatological Association (JDA)
	Japanese Organization of Clinical Dermatologists (JOCD)
	Japanese Society for Investigative Dermatology
	Jordanian Society for Dermatology and Venereology (JSDV)
	Korean Dermatological Association (KDA)
	Kuwait Society of Dermatologists (KSD)
	Lebanese Dermatological Society
	Libyan Society of Dermatology and Venereology
	Maltese Association of Dermatology and Venereology
	Mexican Academy of Dermatology (AMD)
	Mexican Society of Dermatology (SMDAC)
	Mongolian Dermatological Society
	Moroccan Society of Dermatology (SMD)
	New Zealand Dermatological Society Inc. (NZDS)
	Nicaraguan Association of Dermatology
	Nigerian Association of Dermatologists (NAD)
	Norwegian Society of Dermatology and Venereology (NSDV)
	Oman Dermatology Society (ODS)
	Pakistan Association of Dermatologists (PAD)
	Paraguayan Society of Dermatology
	Peruvian Society of Dermatology (SPD)
	Philippine Academy of Clinical and Cosmetic Dermatology (PACCD)
	Philippine Dermatological Society (PDS)
	Polish Dermatological Society (PTD)
	Portuguese Society of Dermatology and Venereology (SPDV)
	Romanian Society of Dermatology (SRD)
	Russian Society of Dermatovenerologists and Cosmetologists (RODV)
	Salvadorian Association of Dermatology
	Saudi Society of Dermatology and Dermatologic Surgery (SSDDS)
	Serbian Association of Dermatovenereologists
	Slovak Dermatovenereological Society (SDVS)
	Sociedad Ecuatoriana de Dermatologia
	Society of Dermatologists, Venereologists and Leprologists of Nepal (SODVELON)
	Society of Dermatovenereology Turkey (TURKDERM)
	Society for Investigative Dermatology USA (SID)
	Spanish Academy of Dermatology and Venereology (AEDV)
	Sri Lanka College of Dermatologists (SLCD)
	Sudanese Association of Dermatology
	Swedish Society for Dermatology and Venereology (SSDV)
	Swiss Society of Dermatology and Venereology (SGDV/SSDV)
	Syrian Arab Society of Dermatology
	Taiwanese Dermatological Association
	Tunisian Society of Dermatology and Venereology
	Turkish Society of Dermatology (TDD)
	UK Translational Research Network in Dermatology (UK TREND)
	Uruguayan Dermatological Society (SDU)
	Venezuelan Society of Dermatology and Dermatologic Surgery (SVDCD)
	Vietnamese Society of Dermatology and Venereology (NIDV)
Allergy and contact dermatitis societies	Dutch Society of Allergology (NVVA)
	European Environmental and Contact Dermatitis Research Group (EECDRG)
	European Research Group on Experimental Contact Dermatitis (ERGECD)
	European Society of Contact Dermatitis (ESCD)
	European Surveillance System on Contact Allergies (ESSCA)
	Irish Food Allergy Network (IFAN)

**Table 2:** List of eczema support (patient) societies to be invited

Name of society
Allergy New Zealand (NZ)
Association Française de l’Eczéma (FR)
Eczema – an Indian Perspective (IDA)
Eczema Association of Australasia (AU)
Eczema Association of Kenya (KE)
Eczema Outreach Scotland (UK, Scotland) Eczema Scotland (UK, Scotland)
Irish Skin Foundation (IE)
Itchy Kids New Zealand (NZ)
La Prévention des Allergies (BE)
Malta Eczema Society (MT)
National Eczema Society (UK)
National Eczema Association (US)
South African National Eczema Association (SANEA) (ZA)
Swiss Allergy Centre (CH)
Talkhealth Partnership (UK)
The Eczema Society of Canada (CA)
Vereniging Mensen met Constitutioneel Eczeem (VMCE) (NL)

Members of the TREAT Research Group will participate in the eDelphi exercise as well as in the final consensus meeting.

### eDelphi questionnaire

The eDelphi questionnaire has been developed using a comprehensive list of domains (i.e. high-level data, e.g. physical examination) and domain items (i.e. more granular data, e.g. blood pressure) identified by the TREAT Research Group through panel discussions and by literature review. For the ‘physician- and patient-reported’ domain and domain items a direct reference was made to guidance from the Harmonising Outcome Measures for Eczema (HOME) initiative (homeforeczema.org). After obtaining an initial list of domains and items, the members of the TREAT Research Group were again asked to review and to add any additional domains and items that they thought should be included. This led to a proposed list of core domains and domain items for the eDelphi questionnaire (Table 3).

**Table 3:** Proposed list of domains and domain items for round 1 of the eDelphi questionnaire

Domains	Domain items
Demographics	Social history
	Marital history
Past AE treatments	Previous topical treatments for AE
	Previous day hospital care treatments for AE
	Previous hospitalisation for AE
	Previous structured education programme for AE
Current AE treatments	Topical treatments
Allergy test results	Delayed contact hypersensitivity patch test
	Atopy patch test
	Double-blind, placebo-controlled food challenge
	Skin-prick testing to foods or aeroallergens
Chronic (inflammatory) comorbidities	Inflammatory bowel disease
	Rheumatoid arthritis
	Diabetes mellitus
Smoking/alcohol/recreational drug history	Smoking history
	Alcohol intake
	Recreational drug history (soft drugs)
Current concomitant medication (i.e. other than specific AE medication	Antihistamines, oral or topical
	Topical antibiotics
	Oral antibiotics
	Allergic rhinoconjunctivitis medication
	Asthma medication
Baseline physical examination	Fitzpatrick skin type
	Weight and height for BMI calculation
	Blood pressure
	Body temperature
	Chest (lung) auscultation
	Heart auscultation
	Lymph node palpation (axillary and inguinal)
	Skin examination
Baseline physician and patient reported domains	Physician-assessed clinical signs, e.g. EASI or SCORAD score
	Investigator/physician global assessment, e.g. IGA
	Patient-reported symptoms, e.g. POEM, itch or sleep score
	Patient global assessment, e.g. PGA
	Generic quality of life score
	Dermatology-specific quality of life score
	AE-specific quality of life score
	Patient-reported satisfaction with AE care received
Baseline investigations and assessments	Blood testing for past/current tuberculosis
	Chest radiograph
	Hepatitis B status
	Hepatitis C status
	Human immunodeficiency virus (HIV) status
	Varicella zoster virus (VZV) immune status
	Pregnancy
	Monitoring P3NP in case of methotrexate use in adults
	Evaluating TPMT level prior to azathioprine use
	Collection of medical photographs to monitor disease extent
Baseline biorepository samples	Collection of blood for biomarkers, e.g. TARC
	Collection of DNA (blood or saliva) for filaggrin analysis
	Collection of biomaterials, e.g. DNA, PBMC or skin biopsy, for a biorepository
Baseline management	Reasons for choosing specific treatment (systemic or phototherapy)
	Routine recording of relative contraindication(s) for selected treatment
Follow-up general questions	Minimum follow-up frequency for registry data entry, once stable therapeutic dose has been reached:
	A. 2 months
	B. 3 months
	C. 4 months
	D. 5 months
	E. 6 months
	Minimum follow-up frequency for registry data entry, after stopping photo- or systematic therapy:
	A. 3 months
	B. 6 months
	C. Annually
	D. No follow-up
Follow-up physical examination	Weight and height for BMI calculation
	Blood pressure
	Body temperature
	Chest (lung) auscultation
	Heart auscultation
	Lymph node palpation (axillary and inguinal)
	Skin examination
Follow-up physician- and patient-reported domains	Physician-assessed clinical signs, e.g. EASI or SCORAD score
	Investigator/physician global assessment, e.g. IGA
	Patient-reported symptoms, e.g. POEM, itch or sleep score
	Patient global assessment, e.g. PGA
	Generic quality of life score
	Dermatology-specific quality of life score
	AE-specific quality of life score
	Reporting of disease control, e.g. flares, fully controlled weeks, by physician
	Reporting of disease control, e.g. flares, fully controlled weeks, by patient
	Adherence to treatment between appointments
	Patient-reported satisfaction with AE care received
Follow-up investigations and assessments	Minimum frequency of safety investigations:
	A. 6 weeks
	B. 8 weeks
	C. 10 weeks
	D. 12 weeks
	E. 14 weeks
	F. 16 weeks
	Collection of medical photographs to monitor disease extent
Follow-up biorepository samples	Collection of blood for biomarkers, e.g. TARC
	Collection of biomaterials, e.g. DNA, PBMC or skin biopsy, for a biorepository

*AE* atopic eczema, *BMI* Body Mass Index, *DNA* deoxyribonucleic acid, *EASI* Eczema Area and Severity Index, *HIV* Human Immunodeficiency Virus, *IGA* Investigator Global Assessment, *P3NP* procollagen type III *N*-terminal peptide, *PBMC* peripheral blood mononuclear cell, *PGA* Patient Global Assessment, *POEM* Patient-oriented Eczema Measure, *SCORAD* SCORing Atopic Dermatitis Index, *TARC* thymus and activation-regulated chemokine, *VZV* varicella zoster virus

Some domains and domain items identified were found obvious by the TREAT Research Group through panel discussions, e.g. age and gender, and will, therefore, not be included in the eDelphi but listed separately (Table 4). This list will be sent to all potential eDelphi participants, attached to the invitation email, and will be made available on the introduction page of the first round of the eDelphi survey. Participants will be asked to confirm whether they feel that any of these domains or domain items should not be automatically included but rather should be included in the eDelphi. In this way, they have the option to add these domains or domain items in round 1.

**Table 4:** List of obvious domains and domain items

Domains	Domain items
Related to baseline ‘demographics and AE’	
Demographics	Date of birth and date of enrolment into registry
	Gender
	Ethnicity
	Educational status
	Current occupation or education
AE diagnosis	How diagnosis AE is established
	Use of validated diagnostic criteria
	Date of onset AE
Past AE treatments	Previous phototherapy
	Previous systemic therapy
Current AE treatments	Phototherapy
	Systemic immunosuppressive therapy
Family history of AE or allergic diseases	
Related to baseline ‘past medical relevant history’	
Allergic comorbidities	Asthma
	Allergic rhinoconjunctivitis
	Atopic eye disease
	Eosinophilic oesophagitis
	Food allergies
	Contact allergies
Other comorbidities	Past malignancies
	Past serious infections
Related to baseline ‘investigations and assessments’	
Baseline safety investigations	Medical history (tuberculosis)
	Full blood count
	Liver function
	Kidney profile
Related to follow-up visits	
Follow up ‘adverse drug reactions’	
Serious adverse events	
Adverse events that cause stop or switch of therapy or change in dosage	
For (serious) adverse events*:* probability of relationship with treatment	
Follow-up ‘management’	
Reason for switching therapy	
Reason for discontinuation of therapy	

*AE* atopic eczema

In addition, patients with AE have critically reviewed the protocol, questionnaire and invitation email concerning content and language to allow patient representatives to take part in the eDelphi in a meaningful way.

### eDelphi survey

The eDelphi questionnaire will be distributed using an online e-management survey system, DelphiManager, maintained by the COMET Initiative. This system has previously been successfully used in several studies to facilitate the determination of core outcome sets [18]. The survey will be pilot tested among members of the TREAT Research Group and AE patients, before going live.

The study will run over three online rounds (Fig. 1). At the beginning of each round, the details of the study with the key objective will be presented. Subsequently, participants will be asked to rate each of the domains and domain items using the GRADE (Grading of Recommendations Assessment, Development and Evaluations) scale, a 9-point scale with 1 to 3 labelled ‘not important’, 4 to 6 ‘important but not critical’ and 7 to 9 ‘critical’ [19]. Participants will have the option of selecting ‘unable to score’ if they feel unable to rate and of providing feedback on a specific item or in general at the end of the survey. Hovering over the text will provide an explanation for key terms.

Participants will be asked to complete each round of the eDelphi exercise and reminder emails will be sent to increase the response rate. To reduce the risk of attrition bias, the importance of completing all eDelphi rounds will be highlighted to all participants at the outset of each round. Participants who save an incomplete questionnaire will be contacted to encourage completion. Furthermore, we will provide clear timelines for the completion of each round and send out personal reminder emails.

### eDelphi round 1

Round-1 content includes (1) demographic data of the participants (age, gender, country of practice, the participants’ stakeholder group, current position (in case of health care professionals), age group of AE patients they predominantly care for (in case of health care professionals), how much experience they have in the care of AE patients (in case of health care professionals), membership of an international dermatology society or AE interest group), (2) the obvious list of domains and their items to be reviewed and potentially commented on, (3) a list of domains with their items to be scored and (4) an option to add any additional domains or domain items.

The total number of registered participants for round 1 will be recorded as the number of participants who have actually completed the rating of the domains and domain items.

### eDelphi round 2

All domains and domain items will be carried through to the second round. Additional domains and items listed by participants will be reviewed by the TREAT Research Group and included in round 2.

In this second round, each participant will be asked to rate the domains and domain items again, together with any additional domains and domain items from round 1. They will be presented with the number of participants who scored and the distribution of scores (%) for each domain and domain item for their particular stakeholder group with a reminder of their own round 1 score. They will be asked to consider responses from the other members of their stakeholder group before rescoring each domain and domain item.

Reasons for changes to scores will be documented by asking participants at the end of round 2 to give a general view of why they changed scores.

Those who have not participated in, or completed, the first round will not be invited to round 2. The total number of invited participants for round 2 will be recorded as the number of participants who have actually completed the rating of the domains and domain items. All domains and domain items will be carried forward to round 3.

### eDelphi round 3

An overview of the domains and domain items that have reached consensus will be presented at the beginning of the round-3 survey.

In this third round, all participants will receive identical feedback, containing the distribution of scores (%) for each domain and each domain item for all stakeholder groups, along with a reminder of their round 2 score. They will then be asked to rescore.

All participants included in round 2 will be invited for round 3. As with the previous rounds, the total number of invited participants for round 3 will be recorded as the number of participants who have actually completed the rating of the domains and items. Results of each stakeholder group will be determined. This will lead to the classification of each domain or domain item as ‘consensus in’, ‘consensus out’ or ‘no consensus’ for each stakeholder group separately, which will be used in the final consensus meeting.

### Consensus meeting

All eDelphi participants will be asked in round 3 of the survey if they are willing to attend the consensus meeting. The meeting will be held face-to-face at a location agreed by the TREAT Research Group and/or by telephone conference within 4 months after the close of round 3. The consensus meeting will be attended by representatives of all eDelphi stakeholder groups. All those who completed the three rounds are eligible. Representation of health care professionals and patients will be of particular importance.

One non-voting neutral participant will act as a facilitator and ensure that the voices of all representative groups are heard, and that the process is not dominated by individual participants.

The nominal group technique will be used [15, 20]. The facilitator will present the results of each round of the eDelphi as a summary of response rates, attrition rates, change in scores in case no consensus is reached and domains or domain items that have reached consensus. The meeting will be held to ratify the domains and/or domain items for which consensus are reached and discuss those where ‘no consensus’ is reached or where there is significant disagreement about importance between stakeholder groups. Consensus results from the eDelphi can only be overturned in this meeting if reasons are very strong and transparent, the same applies for opening discussion around new domains or domain items.

For the domains and domain items that have not reached consensus, voting will take place, ideally anonymised by using electronic handsets and TurningPoint© software to analyse the results in real time.

### Definition of consensus and core set

The definition of consensus for the eDelphi exercise is based on that proposed by the Management of Otitis Media with Effusion in Children with Cleft Palate (MOMENT) study group [21], but amended to take into consideration the multiple stakeholder groups. This definition ensures that the vast majority considers an item to be critically important in the absence of a sizeable minority thinking the opposite. Consensus that a domain or domain item should be included in the core set will be referred to as ‘consensus in’. It will be defined as 70% or more of participants in each stakeholder group scoring its importance as 7 to 9 and less than 15% scoring it as 1 to 3. For ‘consensus out’ it is the other way around, i.e. 70% or more participants scoring as 1 to 3 and less than 15% scoring as 7 to 9. If there is uncertainty about the importance, it will be referred to as ‘no consensus’. Any item where all stakeholder groups confirm ‘consensus in’ will be taken to be in the core outcome set.

The definition of consensus for the consensus meeting is applied as used by the Outcome Measures in Rheumatology Collaboration (OMERACT) group and the HOME initiative [22–24]. Consensus that a domain or domain item should be included in the core set will be defined as less than 30% of the whole group of participants disagrees, i.e. not scoring 7 to 9 (one-sided consensus rule). Instead of using the eDelphi consensus rule for each stakeholder group separately, we will apply this different rule to avoid a small stakeholder group deciding ‘consensus out’ when the rest of the stakeholder groups decide ‘consensus in’.

A core set is defined as a list of variables that are essential to collect for any patient/subject [12].

### Analysis of results

The results of the third and final eDelphi round will be analysed separately for each stakeholder group using the abovementioned definition of ‘consensus in’ and ‘consensus out’. The consensus meeting results will be evaluated for the whole group using the ‘one-sided consensus rule’.

### Data management

Confidentiality of the survey data will be ensured by the use of unique numerical identifiers, anonymously allocated to participants, to ensure unrecognizability of individual responses. Data will be password-protected and accessible only to the TREAT Research Group, whose members will under no circumstances breach confidentiality.

### Ethical requirements

Consent to participate will be assumed if individuals agree to participate and complete the online questionnaires. Ethical guidance has been obtained from the Medical Ethics Review Committee of the Academic Medical Center in Amsterdam (reference number W15_249 # 15.0294). The committee confirmed that the Medical Research Involving Human Subjects Act (WMO) does not apply to this study and that no official approval is required.

## Discussion

By the end of this Delphi study we hope to have reached consensus on the core set of domains and domain items to capture in AE patient registries with a research focus, that collect data of children and adults on photo- and systemic immunomodulatory therapies. This internationally agreed core set has the potential to unify data collection within existing and future AE patient registries to allow direct comparisons and data sharing and pooling. Analysis of these registry data will facilitate insight into the effectiveness, safety and cost-effectiveness of photo- and systemic immunomodulatory therapies across dermatology centres and country boundaries.

Applying the Delphi method has several advantages. First, it allows us to include a large group of international experts and patients who are geographically dispersed to participate. In this way, broad consensus can be obtained. Second, the anonymity of the survey may avoid the dominance of certain persons in contrast to face-to-face meetings. However, this could diminish the positive effects of interaction to be found in face-to-face meetings, which helps to identify reasons for disagreement [17]. Therefore, a final consensus meeting will be held in case of remaining disagreements. Third, feedback is given to participants, allowing them to consider the answers of not only their own but also of other stakeholder groups.

Despite the obvious advantages of the eDelphi process, there are several sources of possible biases. Specifically, there is a risk of disproportionate representation among individual groups, including a dominance of clinicians and researchers, and relative underrepresentation of other groups such as patients and families [14]. By contacting patient organisations and using personal contacts, we hope to enrol sufficient numbers within this important stakeholder group. Dominance of individual participants or representatives of one specific stakeholder group at the face-to-face consensus meeting may be another risk. The deployment of an independent facilitator will minimise this risk by ensuring that all voices will be adequately heard. Furthermore, we have chosen to use a prespecified list of domains and domain items in the survey next to an obvious list suggested by our research group. This may bias responses of participants and overstate the importance of domains and domain items recognised by our research group [25]. However, this was done in an effort to optimise the efficiency and feasibility of the survey. Additionally, participants will have the opportunity to add domains and domain items and to comment on the obvious list in the first round. Another consideration is that patients may struggle to interpret the medical terminology used in the eDelphi, which in turn may limit the value of their response. We will, therefore, involve patient organisations and patients in the development and pilot phase, include help texts in the survey and use plain English where possible. A further potential problem is a declining response rate from round to round [17]. Reminder emails will encourage completion of questionnaires, and we will also only invite people who responded to a pre-Delphi invitation, as they are likely to be more motivated to complete the survey. In addition, the extent of the core set may be a hurdle for patients as they may struggle to tell the difference between what is important to themselves and what is important to be included in a patient registry that is focussed on research. This may encourage them to include more domains and/or domain items than other stakeholder groups or to dismiss domains and/or domain items that they see as unimportant, e.g. surveillance data. This may impact the feasibility of the final core set. Therefore, we will highlight the importance of agreeing on a core set in each round of the eDelphi survey and at the consensus meeting. Another point of debate is the challenge to accommodate potential cross-cultural differences in a core set. We will, therefore, extend our invitation to take part in the eDelphi exercise to colleagues in (East) Asia, Africa, South America, and Australia and New Zealand, in addition to European and North American participant representation (see Tables 1 and 2 with dermatology societies and eczema patient support groups to be invited). Lastly, a core set will only have impact if it is consistently implemented in patient registries with a research focus. To achieve implementation in these registries, we must actively engage with all stakeholders, especially international societies and regulatory bodies after the Delphi exercise is completed to ensure that this core set is accepted and used.

### Study status

The eDelphi study is currently ongoing. After the data of the eDelphi are analysed, a consensus meeting will be planned for defining the final core set of domains and domain items.

## Additional file

Additional file 1: SPIRIT 2013 Checklist: recommended items to address in a clinical trial protocol and related documents. (PDF 199 kb)
Additional file 2: Invitation letter. (DOC 249 kb)

## Abbreviations

AE: Atopic eczema
COMET: Core Outcome Measures for Effectiveness Trials
CRTH2: Chemoattractant receptor-homologous molecule expressed on Th2 cells
EMA: European Medicines Agency
FDA: US Food and Drug Administration
GRADE: Grading of Recommendations Assessment, Development and Evaluations
HOME: Harmonising Outcome Measures for Eczema
ILDS: International League of Dermatological Societies
NICE: UK National Institute for Health and Care Excellence
OMERACT: Outcome Measures in Rheumatology Collaboration
PARENT JA: PAtient REgistries iNiTiative Joint Action
RCT: Randomised controlled trial
TREAT: TREatment of ATopic eczema
