# Supplementary material for: TREatment of ATopic eczema (TREAT) Registry Taskforce: protocol for an international Delphi exercise to identify a core set of domains and domain items for national atopic eczema registries
Source: Trials. 2017 Feb 27;18:87. doi: 10.1186/s13063-016-1765-7 (PMC5330088; doi:10.1186/s13063-016-1765-7)
Supplement: Additional file 2: — Invitation letter. (DOC 249 kb) [file 13063_2016_1765_MOESM2_ESM.doc]

**Additional file 2**

**Invitation letter**
